# A modeling algorithm for exploring the architecture and construction of bird nests

**DOI:** 10.1038/s41598-019-51478-1

**Published:** 2019-10-14

**Authors:** Hadass R. Jessel, Lior Aharoni, Sol Efroni, Ido Bachelet

**Affiliations:** 1 Augmanity, Rehovot, 7670308 Israel; 2 The Mina & Everard Goodman Faculty of Life Sciences, Bar-Ilan University, Ramat Gan, 52900 Israel

**Keywords:** Structural biology, Biological models

## Abstract

The wide variety of nest architectural designs exhibited by passerine birds allowed them to diversify into a wide variety of ecological niches and terrestrial habitats. At present, very little is known about the mechanics of building these structures. Digitizing natural biological structures such as bird nests provides the opportunity to explore their structural properties and behavior under specific conditions by means of computational manipulations, simulations, and analyses. This study describes a generic algorithm for the digitization and exploration of complex interlocked bird nests, and validates it on nests built by the Dead-Sea Sparrow (*Passer moabiticus*) in branches of trees using stiff dry branches. This algorithm takes as input computerized tomographic scans of the nest, identifies and isolates each branch entity within the three-dimensional data, and finally extracts the characteristics of each branch. The result is a reliable three-dimensional digital model of the nest that contains a complete geometric dataset per each of its components, e.g. dimensions and contact points with neighboring components, as well as global properties, e.g. density distribution and network structure. Based on these, we were able to simulate various models of the nest construction process. Altogether, the described algorithm and possible derivatives thereof could be a valuable tool in studying the structure-function relationships of similarly complex biological objects, and may provide further insights into the potential selective mechanisms underlying historical evolution of this distinct nest form.

## Introduction

The field of structural biology is concerned with the relationships between structure and function in biological objects, mainly at the molecular level. Testing specific hypotheses regarding these relationships is typically done by altering the structures using traditional experimental techniques such as genetic mutations, and measuring the consequent behavior of the object under study^1^. However, complex biological structures at the macro-scale level, such as animal made structures, e.g termite mounds^2^, orb webs^3^, and bird nests^4^, that presumably have a genetic basis, are challenging and often impossible to explore this way, since there is no simple injective mapping between genotype and phenotype.

The form and structure of biological objects often reflects their assembly and function. Avian nests are no exception, with a high degree of design variations across families which translates to multiple functionalities. Avian nests are essential for reproductive success, providing a location for rearing the young and safely supporting the incubating bird and its clutch of eggs^5–10^. Different nest designs were shown to provide secondary functions such as defence from parasites or pathogens^11,12^, sexual signalling^11,13^, insulation^14–16^ and crypsis against predation^17^. However, the mechanics of nest building techniques in birds remains considerably understudied^18,19^.

The architectural diversity of nests in passerine birds (order *Passeriformes*) is thought to have played an important role in the adaptive radiation of this group, which now account for more than half of all bird species and occupies nearly all terrestrial ecosystems^19,20^. Studying nest architectures among passerine species provides a mean for understanding nest evolution and the complex behaviour involved in avian nest building in general^21^.

Here we focused on the complex nests built by the Dead-Sea Sparrow (*Passer moabiticus*)^22^ a small passerine of 15–18 g^23^, which currently occupies a highly disjointed range extending from southwest Afghanistan and northwest Pakistan in the east, through Iran, Iraq, Syria, Jordan, Israel, Turkey and Cyprus in the west^24^. The male sparrow establishes the nest structure, 30–50 cm high and 20–35 cm in diameter, weighing up to 1 kg^25^. The massive oval nest is built in tree branches, mainly in *Tamarix* spp. often in river or waterside areas near deserts^26^. The nests are composed of stiff dry branches inserted and interlocked into a complex structure^23,25^. A spiral passage leads from the top of the nest to an incubation chamber lined with soft materials in its lower part^23^ where the female lays and incubates her clutch with a mean clutch size ranging between 3–6 eggs^25,27^.

The Dead-Sea Sparrow’s nest contains mostly branches for construction, but how construction is carried out is unknown. Utilizing branches as a construction material requires certain spatial relationships to ensure that the nest remains attached to the nesting site and retains its structural integrity^28^. Better understanding of the relationship between structure and function in assembled bird nests can be done by breaking down the structure into the elements comprising it and studying how each element affects the architecture as a whole. Recent studies have tried to determine the construction patterns of nests and the factors that affect nest construction both examining the building materials and their mechanical properties^7,29,30^. To date, studying assembled nest constructions is primarily performed by means of deconstruction and characterization of its components to relate the composition of the nest regions to their function^6,7^. Rebuilding the structure for purposes of performing mechanical tests to study its biomechanical properties might therefore be impossible.

Using non-destructive three-dimensional (3D) imaging techniques such as computer tomography (CT) provides the internal structure of the scanned objects for generating reliable volumetric models. Volumetric representations are particularly suitable for exploring the properties and behaviour of the objects under specific conditions, by means of computational manipulations, simulations, and analyses^31–34^. These structures may be 3D printed prior to generating digital manipulations to further explore the structure, e.g by means of mechanical testing^35^. To obtain digital representation from 3D imaging modalities, a computer-based image reconstruction is carried out on a set of two dimensional (2D) tomograms. Digital analysis of the data and 3D models obtained from the actual biological structures provide us with a definitive description of the structures. Moreover, developing *in-silico* nest models based on actual nest structures has made it possible to perform various mechanical tests on the same structure and introduce structural modifications^34^.

To date, detailed description of construction patterns and materials used in avian nests are limited to only a few bird species^6,7,34^. This study improves our understanding of nest architectural designs exhibited by passerine birds by investigating the structure and its potential construction patterns. The goal of this study is to design and implement an algorithm that is capable of mapping the structure of nests made exclusively from tree branches with high accuracy, a relatively complex task due to the intricate multipart structure. The algorithm is validated here on the nests of the Dead-Sea Sparrow. We aimed at specifying each branch in the structure and identifying possible construction patterns by creating digital models of nests, generated by segmenting their CT scans. Defining branch entities was done by identifying the nest structure within the 3D data using image processing and thinning techniques. Finally the characteristics of each branch were extracted and used for statistical analysis of the structure and for generating graphical visualizations and simulations of construction patterns.

## Results

**Figure 1:**
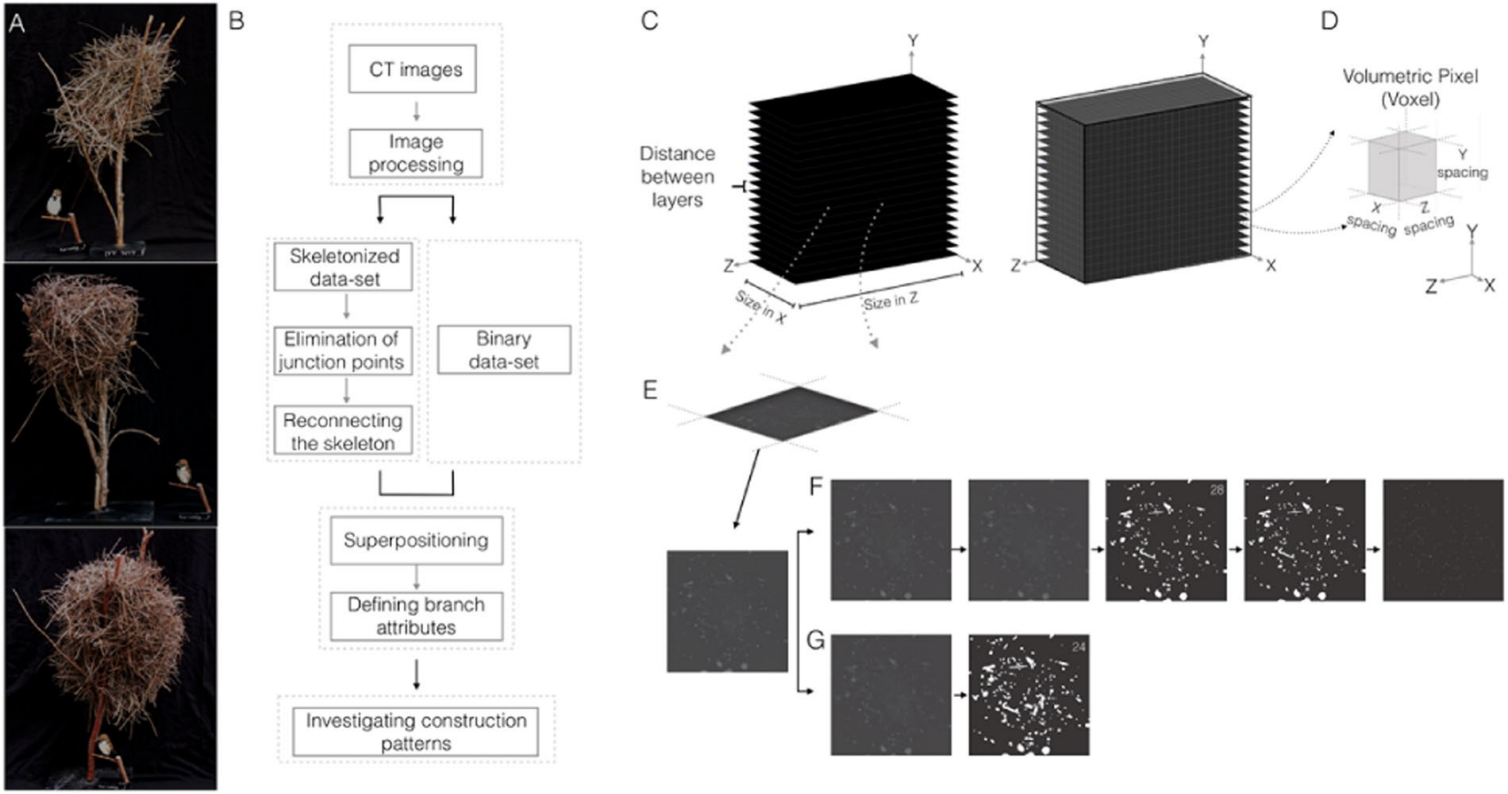
CT image data processing workflow. (**A**) Dead-Sea Sparrow nests. (**B**) Schematic overview of the modeling algorithm. The algorithm first operates on the original CT scans of the nests to standardize and normalize the input. The first stage yields two image types, raw skeleton and binary datasets. A dedicated algorithm is applied to the skeleton 3D model eliminating all junction points between edges, then reconnecting highly correlated edges that originate from the same branch. Superpositioning the binary data set and skeleton is used to calculate branch thickness, length and contact points. Construction patterns of nests can then be investigated (**C**) CT image dataset is generated, showing a voxel intersecting a layer (**D**). (**E**) Layers are processed in parallel in Fiji, (**F**) Showing image processing of grayscale layers; grayscale after neighbourhood averaging 3D filter in the z axis (𝜎 = 5), 3D gaussian blur filter (𝜎 = 1), and global thresholding with a value cut off of 28. Next, a hole filling operation is applied and dataset is skeletonized to identify the three dimensional centreline of the nest structure. (**G**) A second dataset is processed using a gaussian blur filter (𝜎 = 2) and a global thresholding cut off value of 24.

Dead-Sea Sparrow nests are relatively large nests, built by assembling branch components^23^. Collected nests (Fig. 1A) were similar in their overall shape and dimensions (35–45 cm high and 20–25 cm in diameter), which match the measurements reported in older studies^25^. Utilizing only dry branches as a construction material requires certain spatial relationships to ensure that the nest remains attached to the nesting site and retains its physical integrity^28^. For better understanding the nest architecture and these relationships, we generated a pipeline (Fig. 1B, Supplementary Note 1) that generates 3D digital nest models, it identifies and isolates each branch and reveals its parameters (e.g. thickness and length) and the relationships between the branches (e.g. location and contact points).

In order to obtain reliable nest models, nests were scanned by X-ray computed tomography (CT) set to 0.1 mm thick slices with a 512 × 512 matrix of 0.5 mm in-plane resolution, yielding approximately 3700 slices per scan (Fig. 1C,D). The scanning resolution was found to be sufficient for analyzing the individual nest components and identifying the architecture of the nests. These scans revealed the existence of lining material in the incubation chamber and the entrance tunnel leading to it (Fig. S2). The CT scans were used as an input to identify the 3D nest structure and isolate each branch starting with an image data pre-processing workflow (Fig. 1E–G). Pre-processing of the CT images yielded two datasets. The first dataset revealed the 3D skeleton of the structure (Fig. 1F) and the second dataset provided binary images from which branch thicknesses were extracted (Fig. 1G).

**Figure 2:**
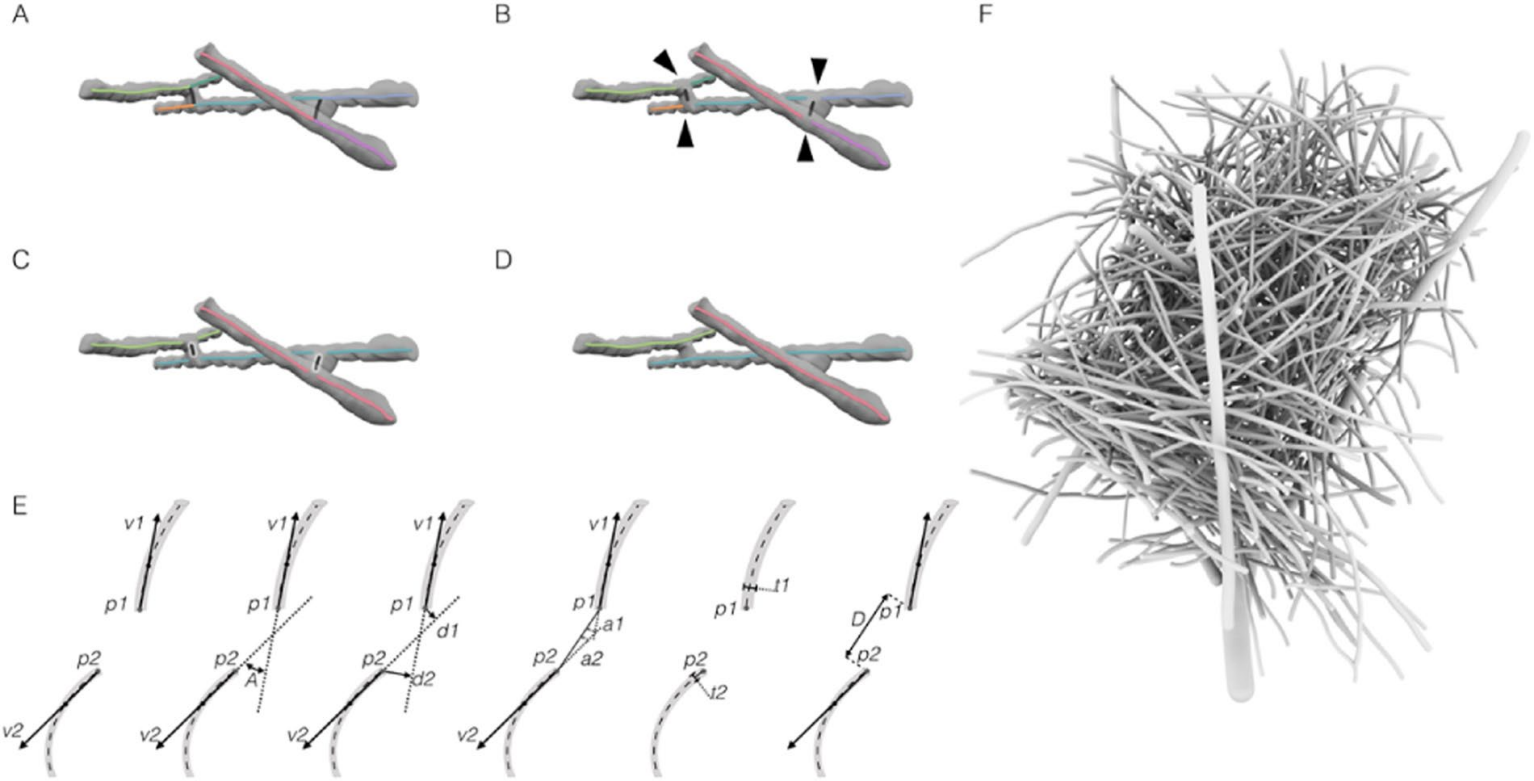
Digitizing an avian nest structure. Illustration example of the process of breaking the skeleton structure and reconnecting highly correlated edges as applied to a sample of branches. (**A**) Structure skeleton with branches represented as edges with different colors (n = 9), including 4 junction vertices and 6 end vertice. (**B**) Breaking the structure by eliminating junction points (black arrowheads), the resulting structure is composed of end-end edges (n = 9). (**C**) Edge groups with high inter-correlation are marked with the same color, with correlated edges linked to each other through the high correlation endpoint couples. (**D**) Short edges representing the contact points are eliminated. (**E**) Illustration showing the different parameters used to measure the correlation between end-points of two branches. Two end-points noted by *p1* and *p2*, The direction of the branch at each end point is measured as a vector from the end point to a point along the branch in a constant length T measure along the branches skeleton. The two direction vectors are noted by *v1* and *v2* respectively. The angle between the two direction vectors is noted by *A*. *A* measurement of the distance between each direction vector and the end point on the other branch is used, the two distances are noted by *d1* and *d2* corresponding to *p1* and *p2*. Measurement of the angle between each direction vector and the vector formed by the two points, noted by *a1* and *a2*. Thickness of each branch at the corresponding end point is noted by *t1* and *t2* respectively. The thickness is measured as the median taken over a fixed number of sample points on the branch’s edge. The distance between *p1* and *p2* is noted by *D*. (**F**) 3D model of reconstructed nest constructed from 739 branches.

In order for this pipeline to generate the most reliable results, the thinning process used to generate the skeleton (branch centerline) did not identify touching branches as separate entities. Rather, in the resulting 3D skeleton, certain branch centerlines were connected with bridges that originate from contact points between branches (Fig. 2A). Therefore, we applied a method designed to optimally analyze the skeleton and break the entire skeleton structure by eliminating all junctions and forks, resulting in simple edges. This process eliminated both *true* and *false* junctions and as a result some *true* branches were broken down into short segments (Fig. 2B,C). To identify topological correspondence between edges (branch segments) that originate from the same branch, a matching algorithm was applied (Fig. 2E). A generalisation of different separation scenarios lies on the relative orientation of the branches and correlation between them. Therefore, various separation and connection scenarios were considered for the development of the matching algorithm, where edges are considered correlated based on the orientation, distances, and branch thickness of endpoints. From the results of this process, highly correlated edges were iteratively linked combining two edges into a single edge (Fig. 2D). This is based on the assumption that highly correlated edges originate from the same branch. The preliminary result of the python code was a list of skeletonized branches, where each branch is described as a set of voxels. Branch thickness was then calculated at each skeleton point by superpositioning the skeleton and the binary image data-sets. For each branch, a median thickness value was finally documented. High quality 3D nest models showing each branch as a separate component were then generated (Fig. 2F), and contact points identified.

**Figure 3:**
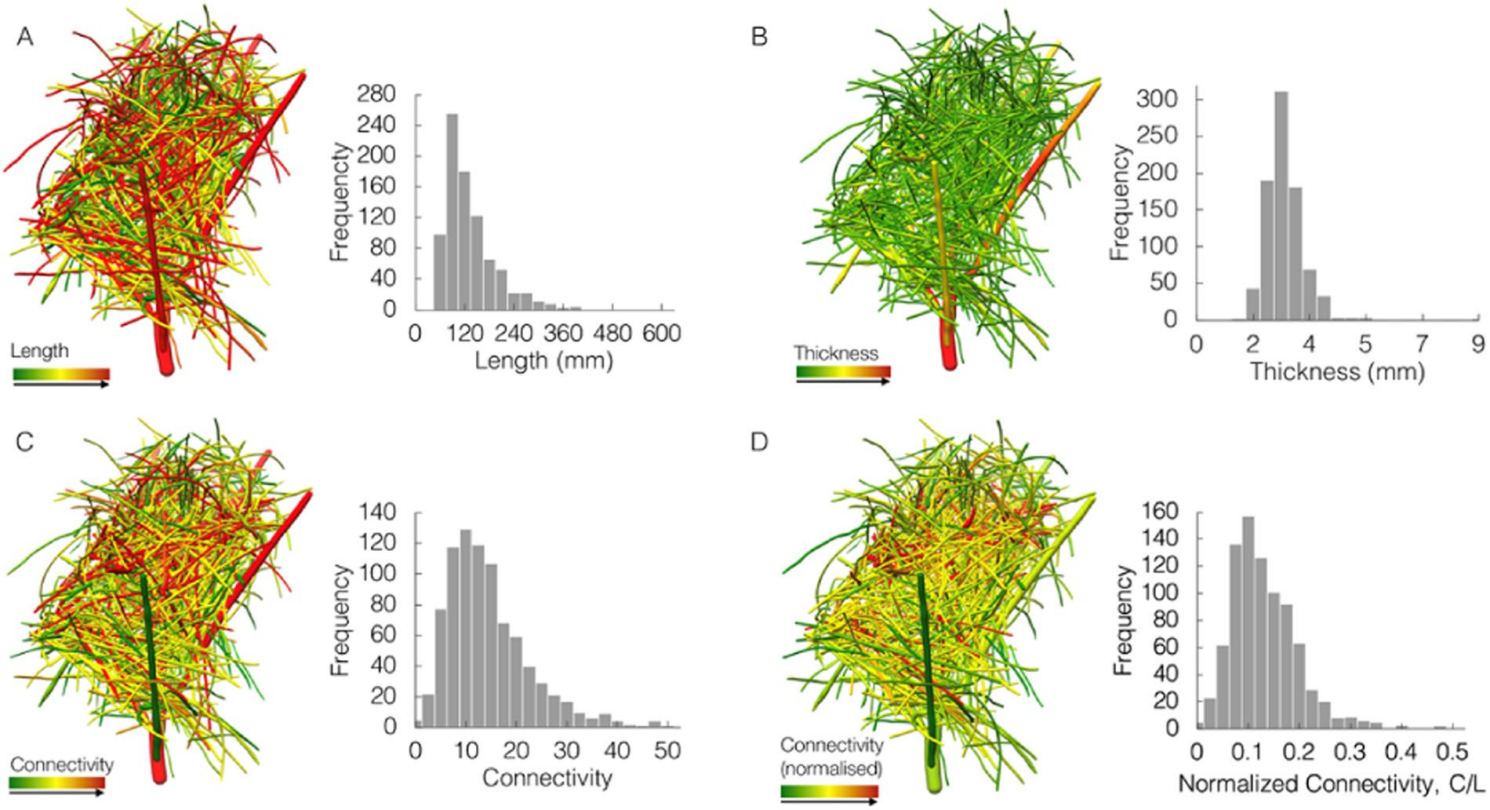
Variation in branch (**A**) length, (**B**) thickness, (**C**) connectivity and (**D**) normalized connectivity in a studied nest. (**A**) Branch length, with low length in green and high in red (left). Frequency of branches for different length values (right). (**B**) Branch thickness, with low thickness in green and high in red (left). Frequency of branches for different mean thickness values (right). (**C**) Branch connectivity, with low connectivity in green and high in red, showing connectivity of each branch in a uniform color (left). Frequency of branches for different connectivity values (right). (**D**) Branch normalized connectivity, with low connectivity in green and high in red, showing normalized connectivity of each branch in a uniform color (left). Frequency of branches for different normalized connectivity values (right).

This analysis revealed, both visually and quantitatively, the distribution of branch length, thickness and degree of connectivity across different regions of the nest (Fig. 3A–D). The positioning of the branches in the nest structure appears to be non-random, with 66% of branches with a horizontal angle greater than 45° and 33% lower, with branches of the exterior nest cup extending to the inner structural wall. There was no significant evidence for discrete categories of length and thickness between the regions of the nest. The birds appear to select branches around a mean length of 159 ± 11 mm and thickness of 2.28 ± 0.33 mm. Branch contact points were distributed randomly throughout the structure, with a mean number of contact points of 30 ± 12 per branch. The branches that originate from the tree, which the nest is constructed upon, are much thicker, longer, and have a higher connectivity value, as visualized in Fig. 3A–D. All these facts integrate to a plausible explanation that the bird constructs the nest mostly by interlocking rather than piling up.

**Figure 4:**
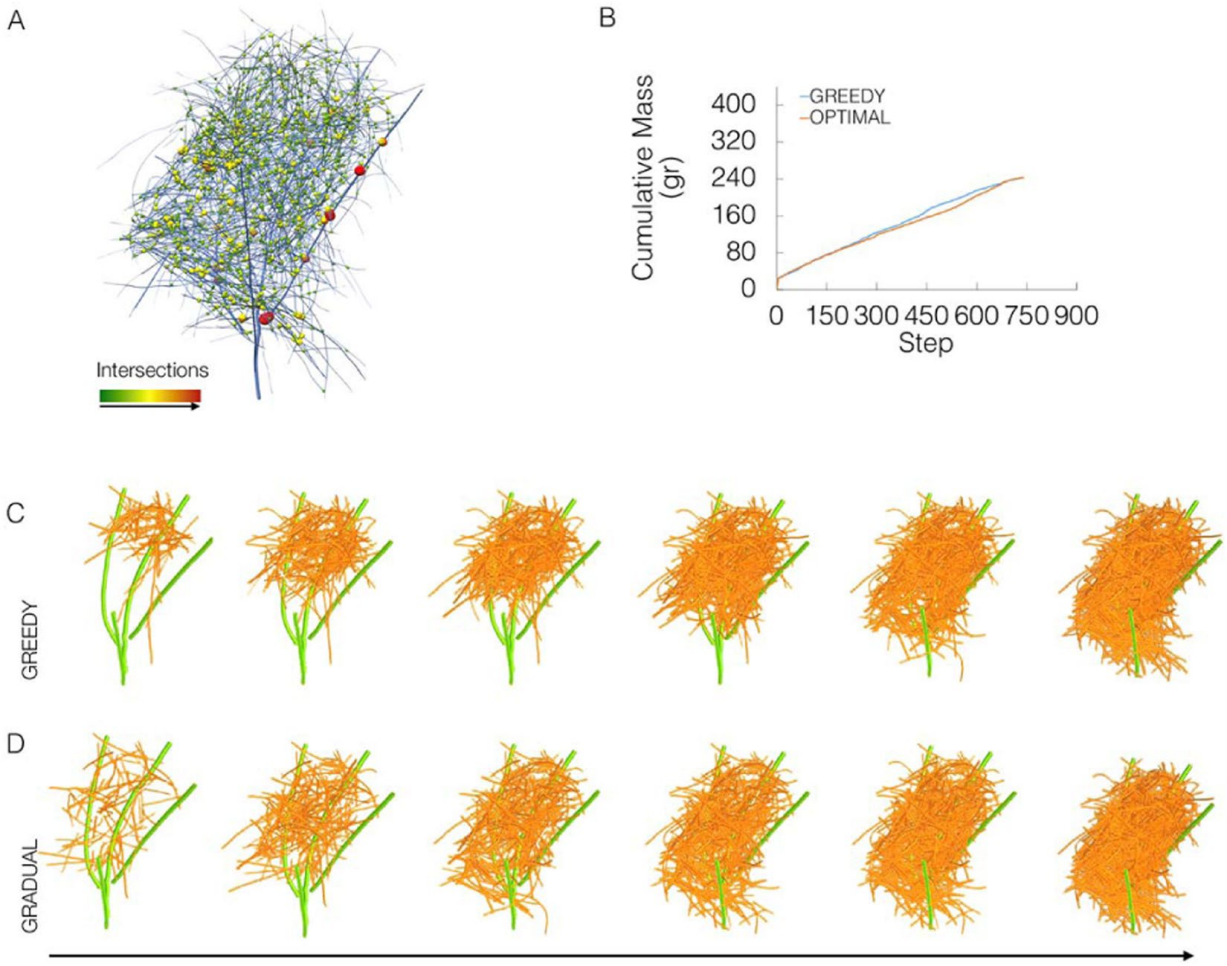
Nest intersection points and construction patterns. (**A**) Skeleton proportional to original thickness (25% of original thickness) showing 208 intersection points as spheres. Color and radius of spheres is proportional to the thickness of branches intersecting. (**B**) Graph represents the cumulative nest mass per step, comparing the two construction strategies. Where in each step an estimated mass of the newly inserted twig is added. (**C,D**) Series of three-dimensional nest models showing two building algorithms to test alternatives for building avian structures. (**C**) ‘Greedy’ algorithm first identifies the structures scaffold (green), then each branch in turn is added, when in each step the branch that is most gravity-connected is added to the existing structure. (**D**) ‘Gradual’ algorithm first builds a minimum viable skeleton that can hold the structure, that is further enriched with branches.

The stability of interlocked nests, constructed using dry branches, depends upon the spatial relationships established between the branches and their geometric properties^36^. In order to infer possible construction strategies from the data, a skeletal map of intersecting points in the nest was first built (Fig. 4A). Two strategies were then devised to simulate most effectively building strategies that correspond to these principles. In the ‘Greedy’ strategy, the magnitude of the link between branches and their relationship to the gravity vector was calculated. In each step, the branch that was the most structurally supported by the already completed structure was added to the existing structure. Alternatively, in the ‘Gradual’ strategy, a minimum viable skeleton was built, that held the structure depending only on spatial relationships between the branches. The interlocking between two branches creates friction. Hence, the force of friction increased in each step with adding new branches to the structure, which increases the pressure between branches and stabilized the structure. From the results of these strategies, each branch was assigned with its estimated mass, and the cumulative nest mass per step was calculated, where in each step an estimated mass of the newly inserted twig was added. (Fig. 4B). According to the simulation results, ‘Greedy’ and ‘Gradual’ reach the final nest in very similar trajectories in terms of cumulative nest mass. However, in ‘Greedy’, the branches in the center of the nest, that have several contact points, are first to be placed, followed by the external branches (Fig. 4C), and ‘Gradual’ dictates a more complex process in which interlocking branches are positioned in the minimal viable skeleton (Fig. 4D).

## Discussion

Dead-Sea Sparrow nests are constructed by the male bird^25^. The nest dimensions recorded here are comparable to those previously described^23^. Many factors can affect nesting success, with the type of the nest being one of them^37^.The massive oval nest was characterized as a domed nest with a top entrance tunnel, as previously reported in Dead-Sea Sparrows^23^ and other passerines^21^. This elaborate domed structure is relatively rare among passerines^19^, having different disadvantages and advantages. Building a large domed nest in trees has the disadvantage of taking longer to construct and greater exposure to sun, wind and weather but the advantage of being less accessible to predators^18^. The design of the Dead-Sea Sparrows nest appears to be optimized in a way that the relatively large domed nest is constructed with an entrance tunnel, as revealed in the CT scan, that largely insulates the eggs from direct sunlight while the nest is still well ventilated^23^. These trade-offs, imply that the environmental and structural parameters have changed over evolutionary time and may have evolved with the emergence of new nest predators or with changing climates and habitat.

Large dome shaped nests are present in other passerine species as well. Certain House Sparrows (*Passer domesticus*) also build similarly fashioned dome nests in trees. House Sparrow nests vary within the species in aspects such as nest location, size, and a broad range of building materials including feathers, grass inflorescences, stalks and roots of plants, barks, threads, strings, and pieces of paper and wool^38^. In warm and dry climates House Sparrows were found to construct elaborate nests in trees with a side entrance^27,39^. This construction method is relatively rare among House sparrows. Investigating the nest structure *in-silico* using the presented algorithm could provide relevant insights into the mechanisms that underlie the construction of a domed nest structure with a side entrance and the morphological evolution of this distinct nest form.

In this study, we presented a new modeling algorithm for digitizing avian structures providing the opportunity to explore their structural properties and reveal relevant insights into the mechanisms that underlie their construction. This pipeline opens up new research avenues given that for many years the quantification and classification of avian nest structures has been subjective and relatively limited^40^. A description of nest construction behaviour is unavailable for Dead-Sea Sparrows and so we are unable to conclusively state the order and strategy birds use when selecting the branches that they place in different parts of the nest. However, the different sizes, location and degree of connectivity of these branch components strongly imply that the process of nest construction involves a specific behaviour in the placement of multiple branch components as construction of the nest progresses. Birds that build nests by means of assembly, collect and join together materials to create a receptacle for the eggs^41^. Assembly techniques can be divided into different construction methods: *piling up*, *molding*, *sticking together*, *interlocking*, *sewing*, and *weaving*. The purpose of the various techniques is essentially to ensure that the nest remains attached to the nesting site and that it retains its physical integrity^42^. Comparing between potential simulated strategies may yield interesting insights into understanding how nests are constructed. The ‘Gradual’ simulation resembles an interlocking strategy in which the structure is supported by a minimal skeleton, yielding a structure in which the nest materials stay together in each step of the building process both because of the spatial relationships established between them through the behaviour of the builder, and because the materials have properties which hold them in that relationship^36^. Whereas the ‘Greedy’ simulation, which presents another building strategy, appears to include both a piling up strategy followed by an interlocking construction method, finally yielding the same nest structure. It may therefore be that construction of a domed nest should not strictly be placed under one construction method, because elements of piling up might be involved as well as behaviour required in constructing the nest.

Computational modeling has greatly augmented our understanding of the role of structure on biological function, and is now a growing strategy for biomechanics in scientific research, with recent advances in model development and simulation platforms leading to growing contributions in scientific and comparative biomechanical studies^43^. The popularity of digital analyses may be due to many factors, from their ability to provide robust quantitative analyses for complex and intricate structures often found in biological systems, to the ability of numerical methods such as Computational fluid dynamics (CFD) and Finite element analysis (FEA) to provide colossal amounts of output data, allowing robust statistical analyses and detailed graphical displays. Digitized nest structures provides the opportunity to simulate and predict how a structure will perform under natural and extreme conditions, and investigate the effect of induced structural modifications on the natural function^28,34^.

Further research into engineering by birds could yield valuable insights concerning the structure-function relationships of similar complex biological objects and provide additional understanding of the adaptation of birds to the environment, and the evolution of particular nest traits^36,44,45^. The algorithm presented in this study, could be used to generate valuable quantitative data for nest materials and yield fascinating insights into the study of avian architectures. The resulting models closely resemble the physical analog, making this process valuable for data analysis and computational biomechanics research. It is thus likely that scientific modeling tools in the future will incorporate a method similar to the one described herein, making it possible for scientists to access, digitally modify and analyze complex natural structures. Furthermore, advanced capabilities to convert CT scans of biological structures into digital data such as the one demonstrated herein may allow for sharing and exploration of diverse biological structures in the scientific community^43^.

As shown here, the data extracted by the algorithm can not only be used to understand the characteristics of bird nests, but also provide a tool to simulate and understand nest building strategies. Additionally, CT scans can be used to distinguish between densities of different woody materials and provide 3D perspective of an object’s interior. While many bird nests are composed entirely out of woody material, other bird nests either use or integrate other collected or self secreted materials for construction^17,40,46^. These nests may be impossible to analyze using the method described in this paper. Hence, other methods will have to be devised for a full analysis of other nest designs. The algorithm presented in this study could be used for generating virtual models of additional types of nests, such as open-cup nests and platform nests. Yet, different building algorithm should be developed for nests that are not constructed on trees as the building algorithms presented herein starts by first identifying and placing the structure scaffold branches that originate from the tree.

Generating methods for digitizing nest structures may provide important insights into avian nest evolution and may suggest potential associations between nest diversification and the adaptive radiations that generated modern bird lineages. The method described and implemented herein points towards new modeling opportunities for which the barriers between the physical and digital domains can be eliminated, facilitating the digital visualization, analysis and manipulation of complex, macro-scale biological structures, such as bird nests.

## Methods

### Nests

Dead Sea Sparrows are small (15–18 g) passerines^23^, that build massive oval nests in tree branches^25^. The male selects the nest site, and establishes the nest structure which consists of stiff dry branches without the aid of any adhesive. This structure has a passage that leads from the top of the nest to an incubation chamber lined with soft materials in its lower part where the female produces a 3–6 egg clutch^25,27^. Three Dead-Sea Sparrow nests, constructed along the Rift Valley in Israel, were obtained from the Steinhardt Museum of Natural History at Tel-Aviv University.

### CT scans

Nests were fixed for scanning in a dual-source CT scanner (Somatom Definition. Flash; Siemens Healthcare, Forchheim, Germany) system. The nests were scanned with the X-ray tube voltage set to 120 kVp and the X-ray tube current of 33 mAs. The best contrast was achieved using a sharp convolution kernel filter (V90 μ) during the scans. X-ray projections were set to an in-plane resolution of 0.5 mm and slice-to-slice separation of 0.1 mm.

### Image processing

CT datasets were imported into Fiji (ImageJ 1.50 g, java 1.6.0_24)^47,48^ an open-source image processing software used for visualization and segmentation. The segmentation process takes advantage of the relatively high contrast in CT images between the nest branches and the surrounding air, and is used to identify and isolate the branches from the background. The following tools and workflow described were applied to all three nests. Image sequence of all CT scanned nest were imported with a pixel spacing value of 0.5 mm in x,y and 0.1 mm in z with a background type of 8-bit unsigned integer. Each dataset was pre-processed using image processing filters, used for noise reduction and averaging. Neighbourhood averaging filter was used with a sigma value of 5 in the z axis. A 3D Gaussian Blur filter was used with a sigma value of 1 in each direction to reduce image noise and reduce detail level. Several segmentation tools were then used to generate two datasets from each scan; one for yielding the structures skeleton and another for extracting branches volumetric data. Since branches used for nest construction appear in a considerable variation of diameters (1.2 mm to 8.8 mm) a range of intensity thresholds were tested for each nest to optimally identify all branches. In each nest, parameters were tested for obtaining the optimal threshold parameters settings fitting the greyscale of the specific CT scan under examination by performing a step of manual validation on a segment of the nest. The optimal threshold cut off value is one that reduces image noise while preserving maximum significant image data (Fig. S3).

As this image sequence was next skeletonized, it was crucial to eliminate inner branch cavities to ensure that the thinning process would not fail to describe the actual geometric structure. Thus, a hole filling operation was applied for filling branches with inner voids or and refining inner branch regions that may have small holes due to local image noise. Finally, a 3D thinning algorithm was applied^49,50^ to obtain the structure’s skeleton, from which the 3D centerlines of individual branches were identified. A thinning function was used to delete border voxels without changing the topology of the nest. The thinning is performed symmetrically and the resulting skeleton is guaranteed to lie in the middle of the cylindrically shaped branch segments. After completion of the thinning step, the skeleton was smoothed by pruning false branches. The skeleton structure was finally exported to an image sequence.

### 3D model generation

An algorithm was applied in python to optimally break the entire skeleton structure into simple edges by eliminating all junctions and forks. The skeleton was first cleaned by pruning tail edges, using an iterative elimination process with a predefined length threshold (T = 5 mm). Next, all junctions were identified and pruned out of the structure by removing pixels (equivalent to 2 mm) off each junction edge, resulting in a simplified structure that contains simple edges only (no forks). As this process eliminates both *true* and *false* junctions, it was necessary to reconstruct the *true* junctions. Various separation and connection scenarios were considered for the development of the algorithm (Fig. S4).

A matching algorithm was then applied to rate the correlation between endpoints *p* of branches oriented in a predefined proximity. To systematically assess whether two segments originate from the same branch, we calculate branch friendship score *C* via

1 $$C=D\cdot A\cdot max(d1,d2)\cdot max(a1,a2)\cdot (\frac{t2}{t1})T$$

Where *D* is the distance between the endpoints, orientation difference *A* is measured as the spatial angle between the orientation vectors *v* with $180-\sphericalangle (v1,v2)$, in which an angle of zero represents a perfect orientation match. The distances *d1* and *d2* are measured between *p1* and *v1, and p2* and *v2* respectively. Angles *a1* and *a2* are measured between the vector $p1\to p2$ and *v1* and *v2* respectively. Finally, the thickness *t* of each branch was recorded. The friendship score is therefore inversely related to the correlation between two endpoints. These parameters often exhibit non-proportional effect on the score, requiring an enforcement of cutoff values before plugging them into the formula. If a parameter’s value is smaller than a certain minimum it is replaced with the predefined value. For *D* and *d* minimum values of 1.0 and 0.5 were enforced respectively. The value enforced on *t2/t1* was 0.1 and *T* is 3. Highly correlated branches were iteratively linked combining two edges into a single edge, and finally short edges were removed. A list of skeletonized branches was generated, where each branch is described as a set of voxels.

The second binary data-set, derived from the pre-processed CT scan, was used to calculate branch thickness. A 3D Gaussian Blur filter was used with a sigma value of 2 in each direction to reduce image noise and reduce detail level. A range of threshold intensities were tested to reveal an optimal cutoff threshold value. The threshold cutoff value was selected by measuring the thickness of several branches and comparing the diameters to scanned images after thresholding, therefore obtaining true thickness values.

Branch thickness was analyzed at each skeleton point, by superpositioning the skeleton and the binary image sets. The measured diameter values were added to each voxel unit. Superpositioning the skeleton and the binary image sets revealed that the pre-processing used to generate the binary images resulted in zero thickness areas along thin branches. As a consequence, we cleaned out branches with a predefined minimum threshold of zero thickness points (T = 10%), and assigned thickness to zero thickness areas by interpolating with neighbouring positive thickness voxel iteratively. Finally, a constant thickness value was documented for each branch by calculating the median thickness of sample points along the branch. Contact points between branches were next identified by measuring the distance between adjacent branch surfaces, as the distance between the skeleton points and branch diameter were known.

### Construction patterns investigation

Rhino-Grasshopper platform was used to visualize the mechanics of building avian architectures, aiming to evaluate the distribution of branches in different stages of the nest building process. Two building algorithms were designed and applied to investigate variations in construction patterns and test alternatives for nest construction. For this, nests were described as a directed network, where the centers of branches are marked as spheres. Spheres size correlates with branches diameter and edges connecting between spheres resemble contact points between branches (Supplementary Note 1). The ‘Greedy’ algorithm automatically identified the contact points from the contact network and assigned each branch with a *gravity-connected* value that is defined by the magnitude of the link between branches and their relationship to the gravity vector. *Greedy* first identified the structures scaffold, usually the thickest branches originally attached to the tree. Each branch in turn was added, when in each step the branch that was most *gravity-connected* (i.e. the most structurally supported branch by the already constructed structure) is added to the existing structure. The ‘Gradual’ algorithm automatically identifies the contact points from the contact network and assigned each branch with two values, *gravity-connected* and *gravity connectivity*. ‘Gradual’ first identified the structure scaffold. Each branch in turn was added, when in each step the branch that was least *gravity-connected* and has *gravity-connectivity* (i.e stays put due to friction with other branches) was added to the existing structure. Building strategies were analyzed.

The data processing was performed on a PC workstation (Intel (R) Xeon (R) CPU E5–2643 v3, 3.4 GHz, 128 GB RAM). Algorithm will be made available upon request.

## Supplementary information

supplementary info
