# Supplementary material for: A modeling algorithm for exploring the architecture and construction of bird nests
Source: Sci Rep. 2019 Oct 14;9:14772. doi: 10.1038/s41598-019-51478-1 (PMC6791838; doi:10.1038/s41598-019-51478-1)
Supplement: Supplementary file 1 — supplementary info [file 41598_2019_51478_MOESM1_ESM.pdf]

A modeling algorithm for exploring the architecture and construction of bird nests

Hadass R. Jessel[1,2], Lior Aharoni[1], Sol Efroni[2], Ido Bachelet[1,3]

1. Augmanity, Rehovot, 7670308, Israel,
2. The Mina & Everard Goodman Faculty of Life Sciences, Bar-Ilan University, Ramat Gan 52900, Israel
3. Address for correspondence: 8 Hamada Street, Rehovot 7670308, Israel. E-mail address: dogbach@gmail.com.

Supplementary Notes

Supplementary note 1: Schematic outline of the pipeline algorithm

Supplementary note 2: CT showing nests' incubation tunnel and chamber

Supplementary note 3: Threshold segmentation for skeletonization

Supplementary note 4: Branch-set scenarios

Supplementary note 5: Building algorithm

Supplementary note 1: Schematic outline of the pipeline algorithm

The algorithm was designed in a pipeline pattern, starting from the image sequences, passing through different filters to achieve the final desired result. In each step and sub-step, the intermediate result was written to a file and read back before the following step to allow easy management of a process that is complex and computationally intensive. **Figure S1** shows a diagram of the entire pipeline, where each sub-step is roughly implemented as a function in the code level. Steps [2] and [3] represent the core of the algorithm, whereas the rest of the steps are more technical and were implemented for complexity reduction purposes and to provide specific information desired in this research. The actual process designed and implemented in a Python script is more detailed and complex. For instance, the entire raster data of both the skeleton and the binary sequences were translated into a dedicated textual format to allow access to the data in a reasonable amount of time, and to lower the complexity of both IO operations and memory consumption. For example, the binary image sequence comprising 3741 images was reduced from 1500MB to a single 89MB file, and the skeleton sequence reduced from 1500MB to 9MB.

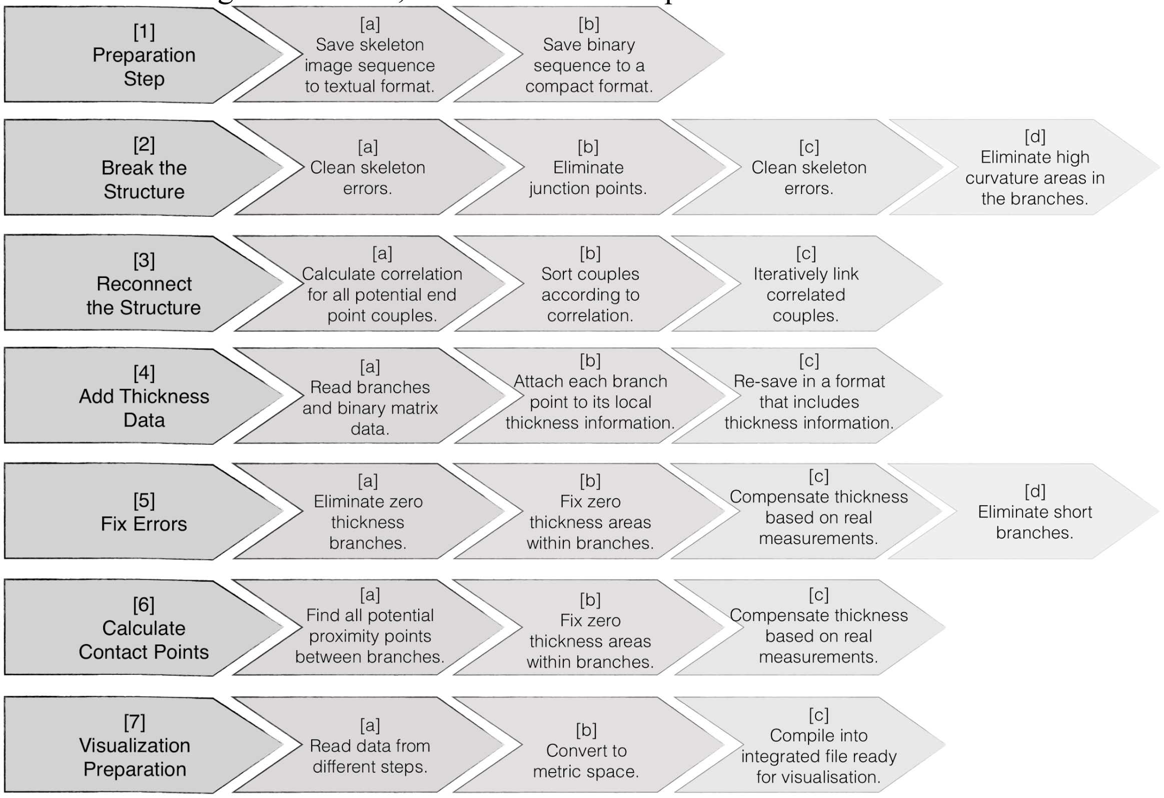

Figure S1: Illustration showing a schematic outline of the pipeline algorithm. Each line from [1] to [7] represents a main step of the algorithm, and a breakdown to sub-steps on the right noted by [a], [b], etc. Sub-steps are correlated with functions implemented in the Python script, excluding reading and loading of the information from/to files between each two adjacent steps. Step [1] is used to translate the raster sequences obtained from FIJI into compact textual representations. Step [2] and [3] represent the algorithm core of breaking and reassembling the skeleton. Step [4] adds the thickness information to the skeleton based on the binary sequence obtained form FIJI. Step [5] is used to fix errors for further calculations. Step [6] is used to analyze the contact points between branches, and finally step [7] is used to translate all the process data into a format that can be plugged into a Grasshopper visualization script described later.

Supplementary note 2: CT showing nests' incubation tunnel and chamber

High-resolution computed tomography shows lining material in incubation chamber and incubation tunnel.

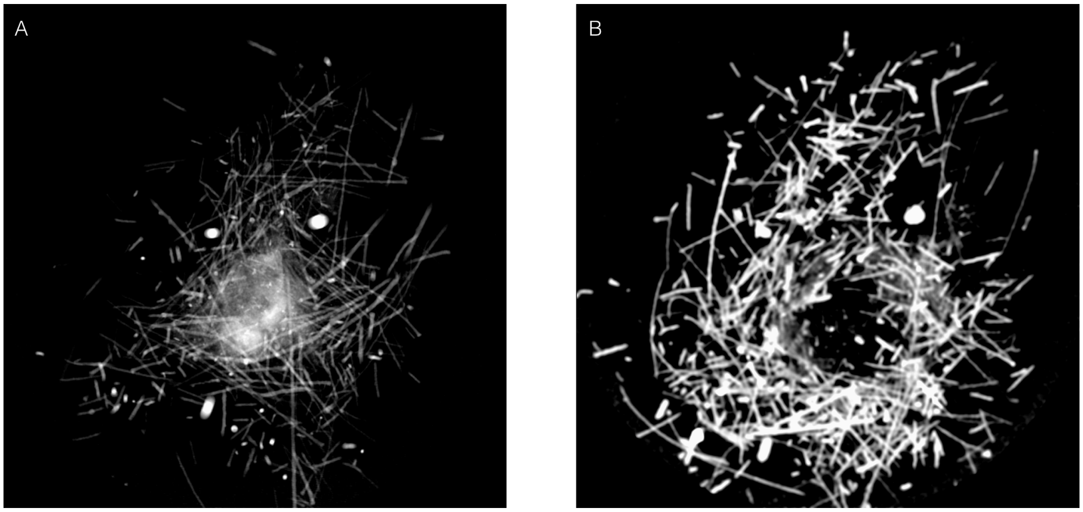

Figure S2: Axial 13.2 mm Thick Slab showing nests' incubation tunnel and chamber. (A) Axial thick slab, mean intensity projection showing lining material in the bottom of the incubation chamber (slab of 128 13.2 mm section). (B) Axial thick slab, maximum intensity projection (MIP) showing incubation tunnel (slab of 128 13.2 mm section).

Supplementary note 3: Threshold segmentation for skeletonization

To enable the thinning process, image data-set was binarized by testing different threshold values. Threshold values are highly dependent on the specific image data-set used, since different CT scanners and scanning parameters may yield sequences with histograms that differ from one another. An optimal threshold is one that reduces image noise while preserving maximum significant image data. In our case the optimal threshold was found to be 28. An optimal threshold can be obtained from testing a single images in the sequence, and finally validated through the entire sequence. In the case that the histogram changes dramatically through the sequence itself, obtaining one optimal threshold for the entire sequence may be very challenging, and more sophisticated approaches may be used. Therefore it is important to control the scanning process to produce high quality sequences in that sense. To explore the optimal threshold of a specific data-set a quantitative approach was used, which included the processing of the data with different thresholds, running it through the structural analysis algorithm described later, and measuring the amount of data lost in each threshold. **Figure S3** shows the count of branches in each length obtained from an image sequence that was processed with different thresholds. Measurements demonstrate a correlation between the amount of data and the threshold used.

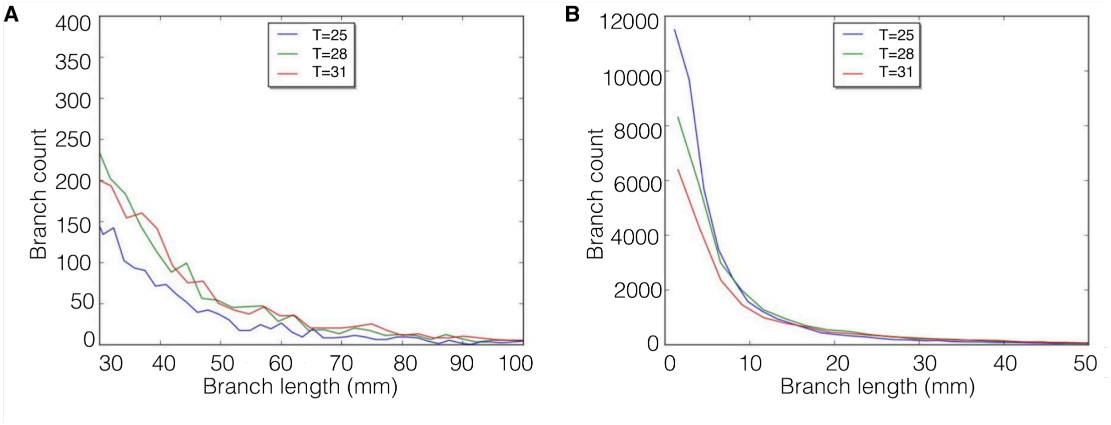

Figure S3: Branch length histogram obtained from different thresholds. Two graphs showing the same plots in different close-ups. (A) Shows the histogram of branch lengths from 30 to 100 mm, and (B) from 0 to 50 mm. Graph (B) shows clearly how raising the threshold value eliminates a large portion of the short branches. The blue plot shows how the low threshold of 25 sacrifices some of the longer branches to clusters that are composed out of shorter branches, i.e. when using T=25, the count of branches in lengths 30-100mm drops dramatically, and the count of branches with lengths 0-5mm rises dramatically, relative to the higher thresholds of T=28 and T=31. T=28 and T=31 show similar behavior in the long branches histogram, however in the range of 5-10mm, T=31 shows dramatic drop relative to T=28, which can be translated as elimination a large portion of meaningful data. Therefore T=28 was chosen as a reasonable tradeoff.

Supplementary note 4: Branch-set scenarios

To fully understand the requirements from a process that separates a skeleton into its original branch components, different separation scenarios were considered. **Figure S4** shows different scenarios of connected and separated version of structures.

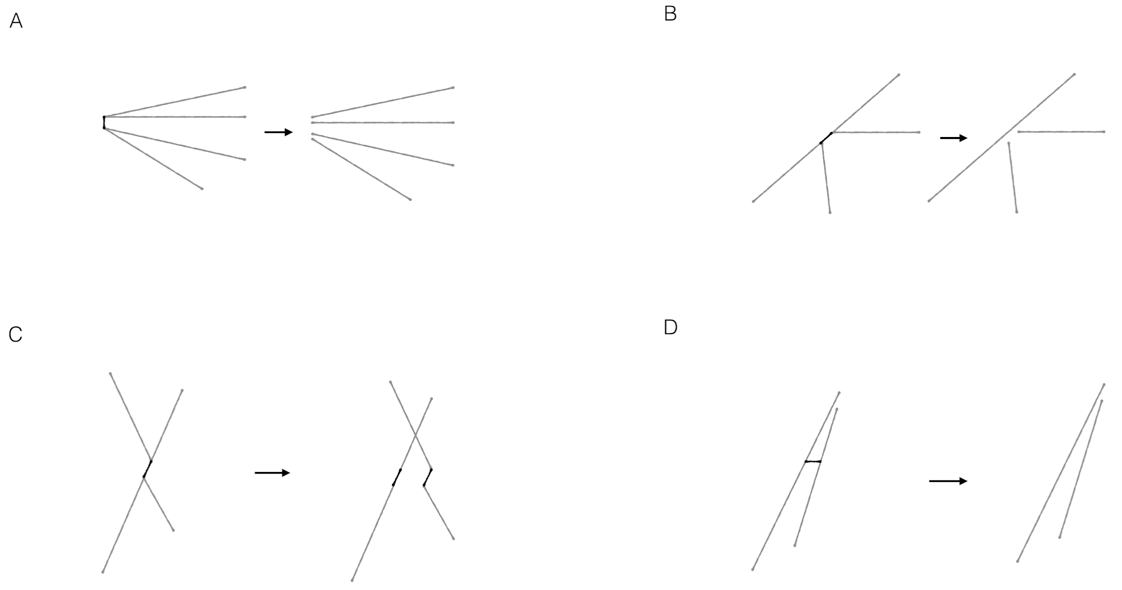

Figure S4: Illustration of branch-set scenarios. Scenarios of connected branches, and their desired separated setting. (A) Four branches connected through one bridge. (B) A bridge resulting from two branches that both meet a third branch, the separated resolution disconnects the two shorter branches and releases the long branch. (C) Two branches crossing each other resulting in a bridge that links between the two halves of both branches and acts as a single part of two different entities. (D) A bridge between two branches with a high proximity point.

Supplementary note 5: Building algorithm

We describe the nest as a directed network, where a link in the network resembles a contact point between two branches. We assume a branch point *P1* on branch *B1* in in contact with point *P2* on branch *B2*. The magnitude of the link between *P1* and *P2* correlates to how much the vector *P1P 2* agrees with the gravity vector direction.

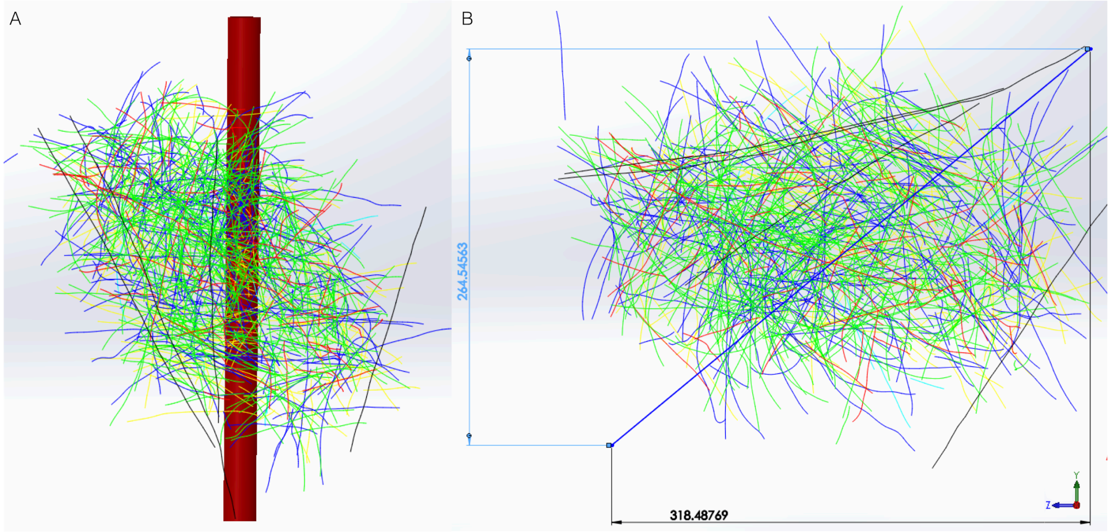

Figure S5: Define direction vector. Branches are plotted to IGES file using grasshopper. Data is opened in Solidworks to draw gravity vector. Vector coordinates are measured and reflected to the algorithm. (X,Y,Z)=(0,264,-318).

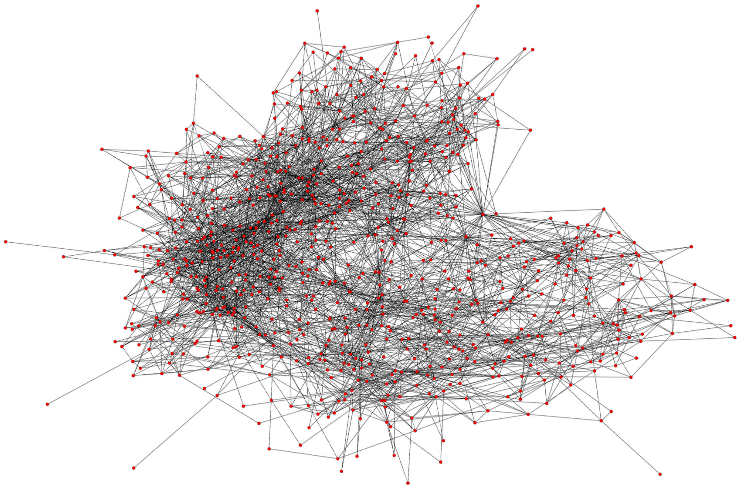

Figure S6: Contact points. Showing branches as red dots, and connections as graph edges.
